# Gut microbiome and health: mechanistic insights

**DOI:** 10.1136/gutjnl-2021-326789

**Published:** 2022-02-01

**Authors:** Willem M de Vos, Herbert Tilg, Matthias Van Hul, Patrice D Cani

**Affiliations:** 1 Human Microbiome Research Program, Faculty of Medicine, University of Helsinki, Helsinki, Finland; 2 Laboratory of Microbiology, Wageningen University, Wageningen, The Netherlands; 3 Department of Internal Medicine I, Gastroenterology, Hepatology, Endocrinology & Metabolism, Medical University Innsbruck, Innsbruck, Austria; 4 Louvain Drug Research Institute (LDRI), Metabolism and Nutrition research group (MNUT), UCLouvain, Université catholique de Louvain, Walloon Excellence in Life Sciences and BIOtechnology (WELBIO), Brussels, Belgium

**Keywords:** intestinal microbiology, obesity, intestinal barrier function, liver, probiotics

## Abstract

The gut microbiota is now considered as one of the key elements contributing to the regulation of host health. Virtually all our body sites are colonised by microbes suggesting different types of crosstalk with our organs. Because of the development of molecular tools and techniques (ie, metagenomic, metabolomic, lipidomic, metatranscriptomic), the complex interactions occurring between the host and the different microorganisms are progressively being deciphered. Nowadays, gut microbiota deviations are linked with many diseases including obesity, type 2 diabetes, hepatic steatosis, intestinal bowel diseases (IBDs) and several types of cancer. Thus, suggesting that various pathways involved in immunity, energy, lipid and glucose metabolism are affected.

In this review, specific attention is given to provide a critical evaluation of the current understanding in this field. Numerous molecular mechanisms explaining how gut bacteria might be causally linked with the protection or the onset of diseases are discussed. We examine well-established metabolites (ie, short-chain fatty acids, bile acids, trimethylamine N-oxide) and extend this to more recently identified molecular actors (ie, endocannabinoids, bioactive lipids, phenolic-derived compounds, advanced glycation end products and enterosynes) and their specific receptors such as peroxisome proliferator-activated receptor alpha (PPARα) and gamma (PPARγ), aryl hydrocarbon receptor (AhR), and G protein-coupled receptors (ie, GPR41, GPR43, GPR119, Takeda G protein-coupled receptor 5).

Altogether, understanding the complexity and the molecular aspects linking gut microbes to health will help to set the basis for novel therapies that are already being developed.

## The human gut microbiome

**Figure 1:**
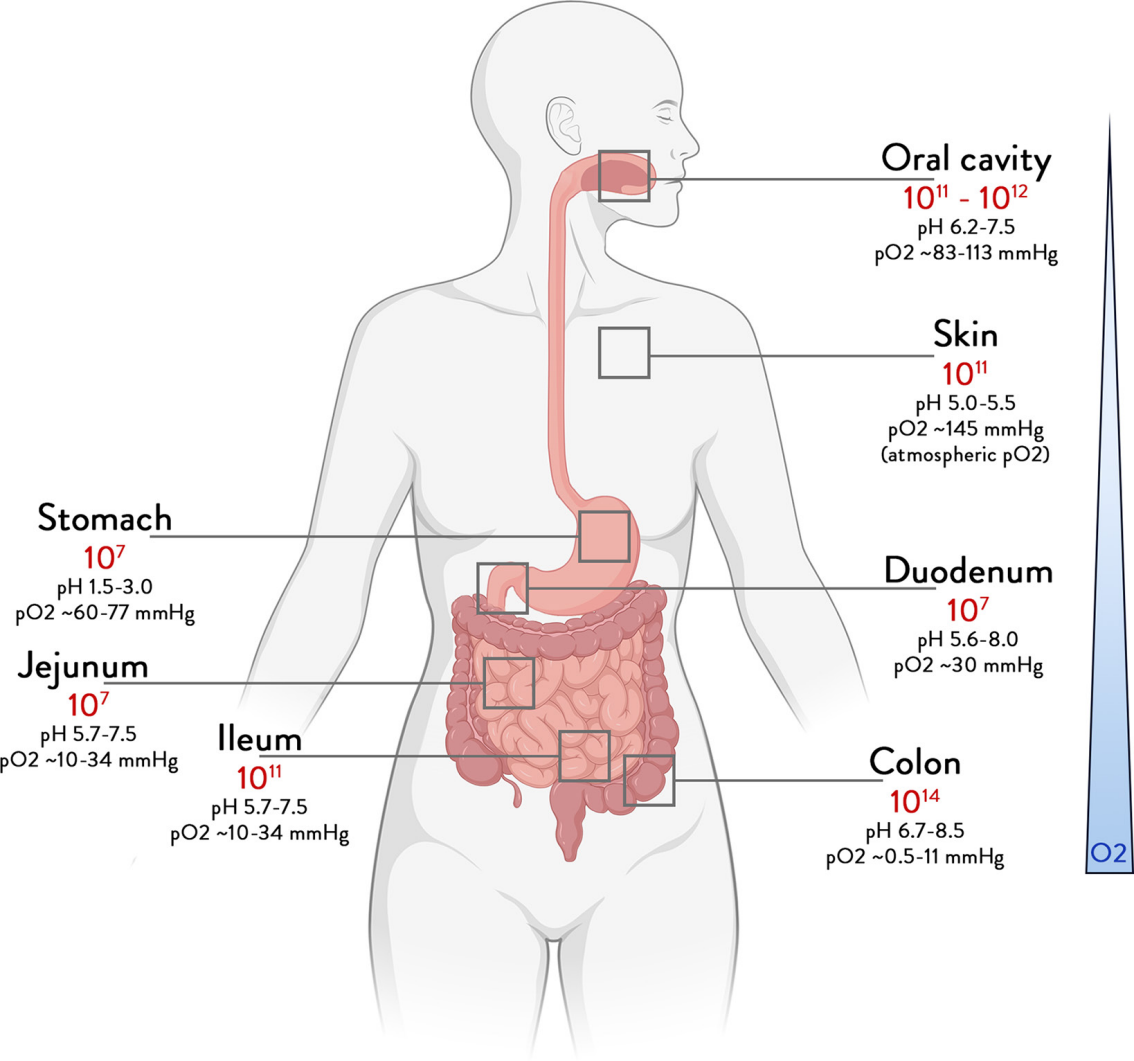
Total abundance of bacteria according to the different body sites. Bounds for bacteria number in different organs, derived from bacterial concentrations and volume.^190 191^

The human microbiome is considered here as the collection of microbes, their genes and their products that colonise our body since birth and are transferred vertically.^1 2^ While all body sites are colonised (figure 1), the highest microbial numbers are found in the gut that has been studied extensively.^3^ Here, we review the main and most recent findings that address the way gut microbes, their activities and mediator molecules can contribute to our health.

In healthy subjects, the oral and saliva microbiomes contain millions of microbes that are swallowed daily with our food, but their persistence in the gut is impeded by many factors, including the acidity of the stomach, the production of bile acids (BAs), digestive enzymes and antimicrobial proteins in the duodenum and beyond. A great number of other major variables affect further downstream microbial colonisation, such as chemical parameters like pH, oxygen concentrations and redox potential, the biological production of mucus, bile and antibodies, as well as physical aspects, including gut architecture, peristalsis and transit times (figure 1). Hence, a concentration gradient of microbes is found along the small intestine, as microbial abundance in duodenal aspirates were found to be a 1000-fold lower than that of oral samples, although consisting of somewhat similar microbial taxa.^4^ Consequently, the small intestine contains an increasing number of thousands to several hundred million of cells per gram of content with partly oxygen-tolerant Firmicutes and Proteobacteria as major phyla.^5 6^ This all culminates in the lower gut where climax communities of up to 100 billion cells per gram reside for up to a few days, since transit in the colon is over a dozen times longer than that in the small intestine. Hence, the colonic microbiome is dominated by mainly anaerobic bacteria, including thousands of species and millions of genes, distributed among the major phyla of Firmicutes (predominantly Ruminococcaceae and Lachnospiraceae), Bacteroidetes, Actinobacteria, Proteobacteria and Verrucomicrobia (*Akkermansia*)^7–10^ (figure 1). Excreted as faeces, it is this biomass that makes up what is usually termed the gut microbiome that has been associated with a plethora of diseases and is highly modifiable by diet and drugs (table 1). It provides the starting material for faecal microbiota transplantation (FMT) that has been shown to cure patients with recurrent *Clostridioides difficile* infections and other diseases.^11–15^

**Table 1:** PubMed-listed articles regarding topics, “microbiome and diseases”

Diseases	PubMed search	PubMed search
	“disease & microbiome”	“disease & microbiome/clinical trial”
IBDs	2867	36
Coeliac disease	524	20
IBS	1516	96
Colorectal carcinoma	1525	43
Liver disease	4927	113
Pancreatic disease	766	20
Obesity	7146	292
Type 2 diabetes	2155	99
Non-alcoholic fatty liver disease	1383	31

PubMed search 15 December 2021.

It is important to note that we can live without a colon but not without a small intestine that features the largest mucosal surfaces of our body where our food is further digested and taken up, contains most of the gut receptors, immune and nerve cells and is increasingly implied in essential microbe-host crosstalk. While hard to approach experimentally, a variety of new technologies have been developed in recent years, which include catheters or capsules to sample, deliver or inspect.^16–18^ In addition, small intestinal effluent obtained from ileostomies was studied and found to contain up to 100 million microbes per gram wet weight that formed personalised communities, showing day and night rhythms reflecting food intake and processing.^19^ Functional (transcriptomics and targeted metabolomics) and metagenomic analysis of such samples revealed the colonising *Streptococcus* and *Lactobacillus* spp to express a large reservoir of highly effective transport systems that compete with the host for sugar uptake and use, generating lactate and acetate that are substrates for *Veillonella* spp and are converted then into propionate.^20^ Recent studies using specially developed catheters confirmed these communities and revealed that the duodenal microbiota exhibited higher compositional dynamics correlating with the pH as compared with the jejunum, which is the intestinal compartment with the largest surface and is responsible for most sugar, protein and lipid digestion and absorption.^5 21^ While there is a continuum between the duodenum and jejunum, the more proximal ileum has a large mucus layer, reminiscent of the colon, and is colonised with several anaerobes, including members of the *Bacteroidia,* Ruminococcaceae and Lachnospiraceae, some of which are also implied in BAs transformation (see section bioactive lipids/bile acids).^21 22^

The duodenum and its microbes have emerged as a major factor in a variety of metabolic and possibly immune diseases.^23^ Support for this and further new insight derived from duodenal delivery of FMT that alleviated symptoms of metabolic syndrome or autoimmune disease.^24–26^ Moreover, duodenal perfusions of live or dead *Lactobacillus* spp have been found to affect the host immune response, providing an experimental system for human discovery.^27–29^ This has been recently exploited for the analysis of a single duodenal dose of *Anaerobutyricum soehngenii* (previously known as *Eubacterium hallii*)^30^ that increased the duodenal expression level of the gene for regenerating islet-protein 1B almost 10-fold and also increased serum glucagon-like peptide-1 (GLP-1) and secondary bile salts in metabolic syndrome subjects, thereby potentially explaining their improved response to glucose.^31 32^

Although there is an evident vertical gradient in the gut, a horizontal gradient also exists and has been studied most extensively in the colon. Importantly, there exist oxygen, redox and mucus gradients that starts at the mucosal surface and stretches to the lumen, resulting in architecture of the microbial communities.^33^ Broadly speaking, these start with mucus-degrading consortia that are usually dominated by the mucolytic and microaerophilic *Akkermansia muciniphila* and end with strictly anaerobic communities, including butyrate-producing and propionate-producing Ruminococcaceae, Lachnospiraceae and *Bacteroidia* as well as homoacetogens and methanogens that convert hydrogen and carbon dioxide into acetate or methane, respectively.

## The gut microbiome and various intestinal and extraintestinal diseases

The gut microbiome has been associated with several intestinal and extraintestinal disorders.^34^ Many large studies investigating the gut microbiome and its relevance have been performed in specific gastrointestinal (GI) disorders such as intestinal bowel diseases (IBDs),^35^ coeliac disease,^36^ irritable bowel syndrome (IBS),^37^ colorectal cancer (CRC),^38^ chronic liver diseases^39 40^ or pancreatic disorders.^41 42^ IBDs, prototypic inflammatory disorders of the intestine, are associated with deviating gut microbiome composition and indeed facultative anaerobes outgrow have been reported, especially in the context of active inflammation and metabolite disturbances including BAs, short chain fatty acids (SCFAs) and acylcarnitine pathways.^35^ Longitudinal analysis in infants at risk for coeliac disease, another frequent inflammatory intestinal disorder, demonstrated an increased presence of several microbial species such as *Dialister invisus, Parabacteroides* spp or Lachnospiraceae and certain metabolites such as tryptophan metabolites before disease onset whereas various anti-inflammatory strains such as *Faecalibacterium prausnitzii* or *Clostridium clostridioforme* were decreased.^36^ IBS, a frequent functional disorder of the GI tract, has been associated with IBS subtype-specific changes in the gut microbiome and related metabolites, with purine metabolism being especially affected.^37^ CRC, the most common malignancy in the lower gut, has been convincingly correlated with a disturbed gut microbiome and implicated certain bacteria such as *Fusobacterium nucleatum, Escherichia coli* or *Bacteroides fragilis*, some of which are derived from the oral microbiome.^38^ Chronic liver diseases, especially advanced liver diseases such as liver cirrhosis, are characterised by profound microbial aberrations and data from interventional studies with prebiotics, probiotics and antibiotics have well established that the gut microbiome plays a key role in these diseases.^40^ Pancreatic adenocarcinoma, an increasingly recognised malignancy in the Western world, has also been linked to an impaired gut microbiome as intratumoral microbiome composition affects the host immune response and natural history of the disease.^42^

The gut microbiome has been extensively investigated in the past years in obesity and obesity-related disorders such as type 2 diabetes (T2D) and non-alcoholic fatty liver disease (NAFLD). Many studies have tried to link an altered gut microbiome to obesity and indeed interventional studies with certain bacterial strains such as *Akkermansia muciniphila* have shown effects on obesity-related parameters.^43^ T2D has also been characterised by an impaired gut microbiome in Asian and European populations.^44 45^ In T2D, microbial variations were strongly correlated with the presence of insulin resistance and several studies implied that the gut microbiome affects glucose regulation.^46^ NAFLD is currently the most common chronic liver disease in the Western world and is considered a prototypic metabolic disorder at the interface of obesity, metabolic syndrome and T2D. There is growing evidence that the gut microbiome-liver axis plays a role in NAFLD, especially in cases of fibrosis and progression towards more advanced disease stages, such as non-alcoholic hepatic steatosis.^47^ Several studies have now demonstrated that NAFLD is characterised by a bloom in certain Enterobacteriaceae, *E. coli* and a decrease in *F. prausnitzii*. Recent data also suggest that in NAFLD microbiome deviations and instability may exist over many years and might even precede development of NAFLD and T2D.^48^ There is a growing number of GI and metabolic disorders where the gut microbiome has been investigated (see table 1 for an overview).

## Gut microbes and metabolic disorders: molecular actors

**Figure 2:**
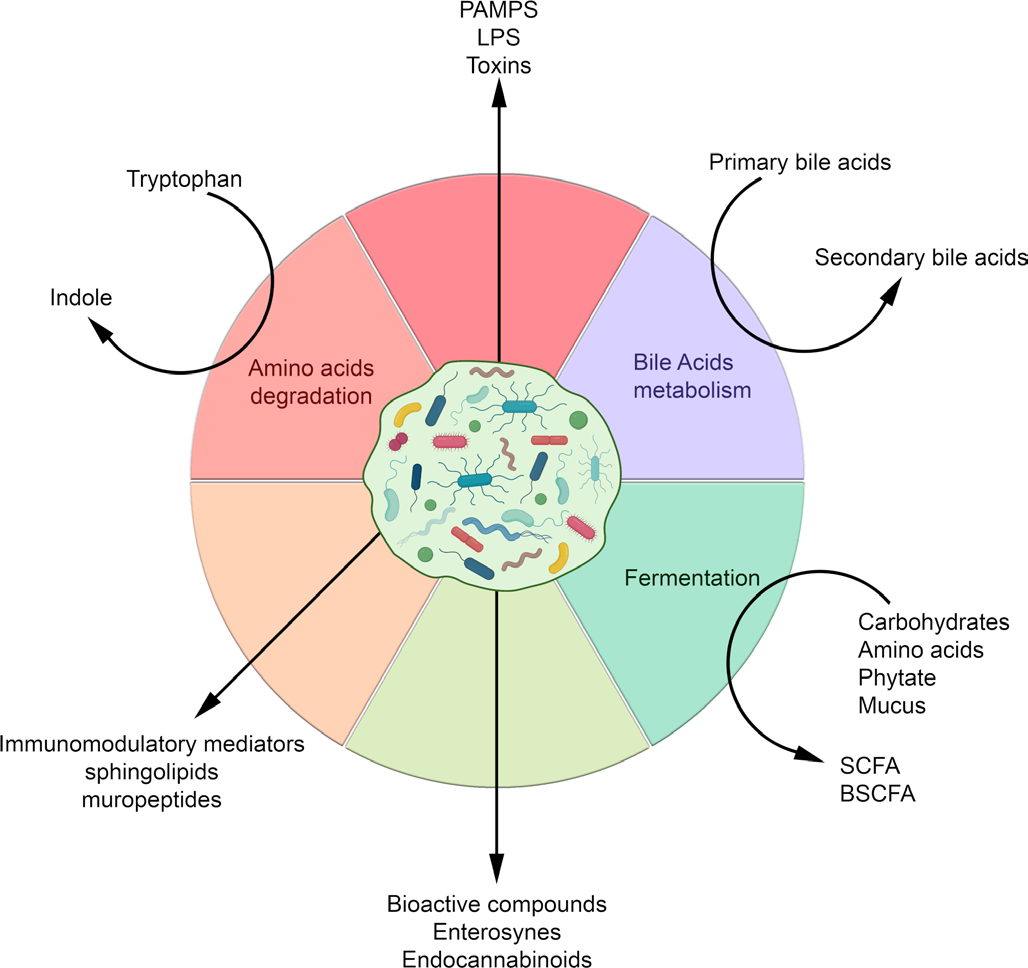
Molecules and metabolites produced by the gut microbiota according to the nutrients or metabolic source and their derived compounds. BSCFA, branched SCFA; LPS, lipopolysaccharides; PAMPs, pathogen-associated molecular patterns; SCFA, short chain fatty acids.

The gut bacterial community plays an important role in the regulation of multiple aspects of metabolic disorders. This regulation depends, among other things, on the production of a wide variety of metabolites by the microbiota and on their interactions with receptors on host cells that can activate or inhibit signalling pathways, and either be beneficial and detrimental to the host’s health (figure 2).

The bacterial metabolites involved in these interactions are very diverse and range from small molecules to large macromolecules. They include by-products of bacterial metabolism, such as SCFAs, and complex macromolecules necessary for bacterial integrity, such as peptidoglycan and lipopolysaccharides (LPS) (figure 2).

The abundance and availability of these metabolites are dependent on the microbial composition and are therefore subject to modulation by diet and environmental factors.^49–51^ The main molecular actors are discussed below.

### Short chain fatty acids and impact on host health: molecular mechanisms

**Figure 3:**
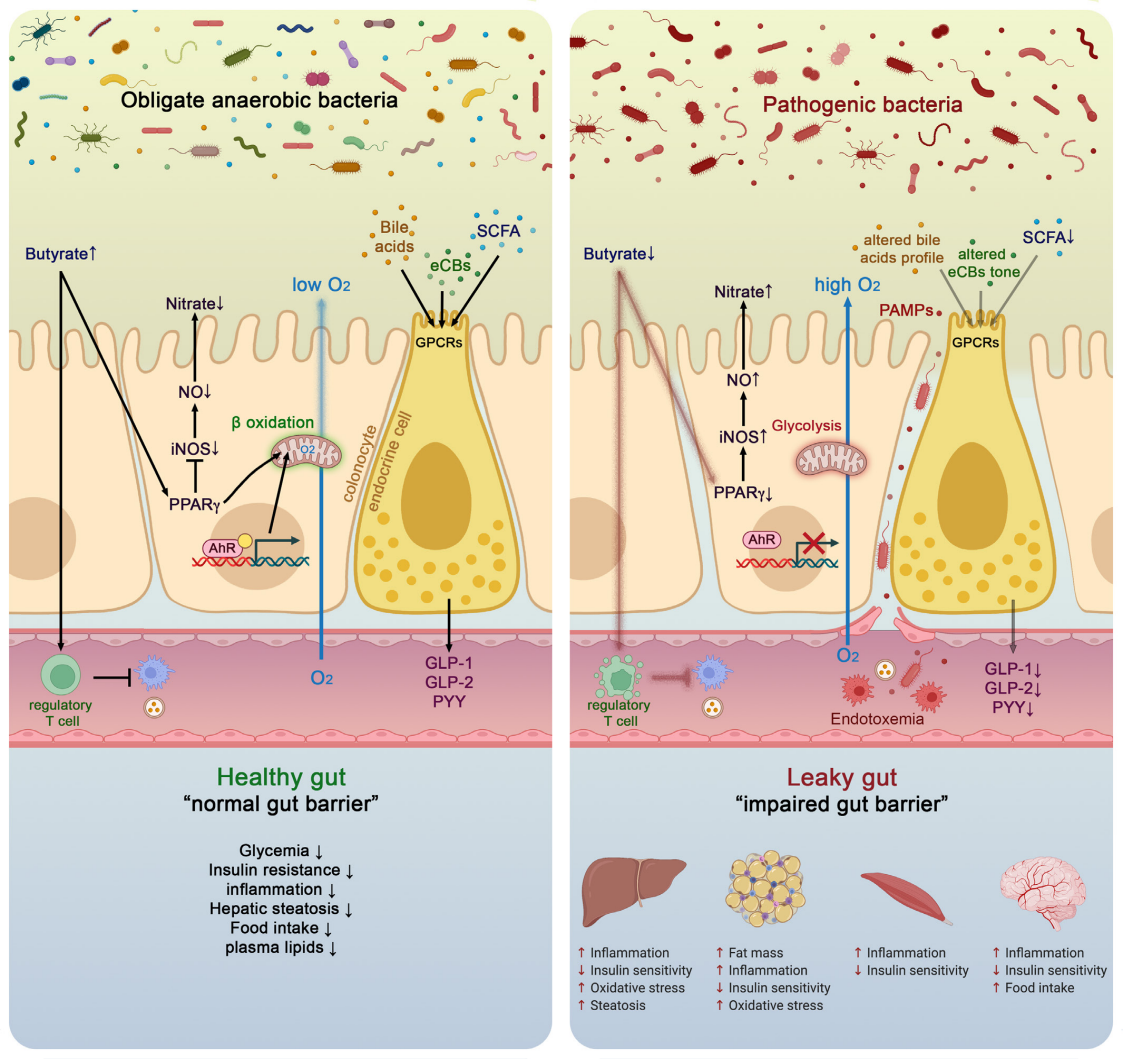
Molecular mechanisms linking gut microbiota and host health in both healthy and pathological situation. In healthy situation, colonocytes use butyrate as energy substrate via the beta-oxidation in the mitochondria, thereby consuming oxygen and directly contributing to maintain anaerobic condition in the lumen. Butyrate also binds to peroxisome proliferator-activated receptor gamma (PPARγ) which in turn repress inducible nitric oxide synthase (iNOS), decreases nitric oxide production (NO) and eventually nitrate production. Conversely, in pathological situations low butyrate content in the lumen is associated with lower PPARγ activity, increased glycolysis and lower oxygen consumption. This is associated with a higher expression of iNOS which in turn produces more NO and eventually increases nitrates availability for specific pathogens. Butyrate can also stimulate immune cells such as regulatory T cells (Treg) to reduce inflammation. The nuclear transcription factor aryl hydrocarbon receptor (AhR) is highly expressed and activated in healthy colonocytes, whereas agonists of AhR are lower or reduced AhR activity can lead to altered gut barrier function. Enteroendocrine cells (L-cells) are expressing several key receptors activated by short chain fatty acids (SCFAs), specific endocannabinoids (eCBs) and bile acids (BAs). Activating these receptors increase the secretion of key gut peptides such as glucagon-like peptide (GLP)-1, GLP-2 and peptide YY (PYY). Altogether, the interaction between the gut microbes and these molecular actors contributes to reduce intestinal permeability, to improve insulin secretion and insulin sensitivity, to reduce food intake, to lower plasma lipids and to avoid hepatic steatosis and metabolic endotoxaemia. All these effects are associated with lower inflammation. Conversely, opposite effects have been observed in pathological situations.

The small intestine is highly specialised in the breakdown, emulsification and absorption of nutrients and few nutrients will escape digestion. In normal conditions, for example, <5 g/day of fat will reach the colon. The same principle is true for the digestion and absorption of simple carbohydrates (broken down into sugar molecules) and most proteins (converted into amino acids), although, depending on the level of intake, some proteins will reach the colon. Conversely, complex carbohydrates, such as dietary fibres are non-digestible, meaning that the body lacks the necessary enzymes to digest them allowing them to escape digestion in the small intestine. In the colon, however, they can be used as an energy source by specific resident bacteria. Various gut microbes will contribute to the metabolisation of these non-digestible carbohydrates into different SCFAs molecules (eg, acetate, butyrate and propionate). SCFAs are chemically well-characterised and their impact on health has already been extensively documented.^52–54^ These compounds regulate numerous metabolic pathways in the gut and at distance such as in the liver, the adipose tissue, the muscles and the brain (figure 3).^55–57^ Nowadays, these microbial metabolites are known to contribute to numerous physiological effects ranging from the modulation of energy homeostasis, glucose/lipid metabolism, inflammation and even immunity and cancer.^52^

Thanks to a series of experimental studies, many of the molecular mechanisms by which a diet enriched with fermentable dietary fibres (eg, prebiotics) are able to decrease body weight gain, fat mass development, insulin resistance and energy intake have been discovered.^58–62^ Among them, it was found that modulating the gut microbiota using prebiotics led to a higher endogenous production (ie, mRNA and peptides) and portal vein secretion of several gut peptides produced by the L-cells such as GLP-1, GLP-2 and peptide YY (PYY) (figure 3).^59–61 63–65^

These effects are not exclusively limited to one type of fermentable carbohydrates since the microbial fermentation of resistant starches or arabinoxylans into SCFAs produces similar physiological effects linked to increased plasma GLP-1 and PYY levels.^66–69^ However, the chemical structure of the fermentable fibres is directly related to the SCFA production profile since the quantity of butyrate, acetate or propionate generated will depend on the type of fibres. For example, inulin is described as propionogenic, whereas resistant starches are more butyrogenic. Of note, several colonic bacteria use alternative pathways to also produce butyrate from amino acids such as lysine or propionate from plant compounds such as phytate.^70 71^ It is worth noting that the sources of SCFAs are derived from the diet and they can originate from the host itself via the fermentation of the intestinal mucus that covers the intestinal epithelial cells.^72^

**Figure 4:**
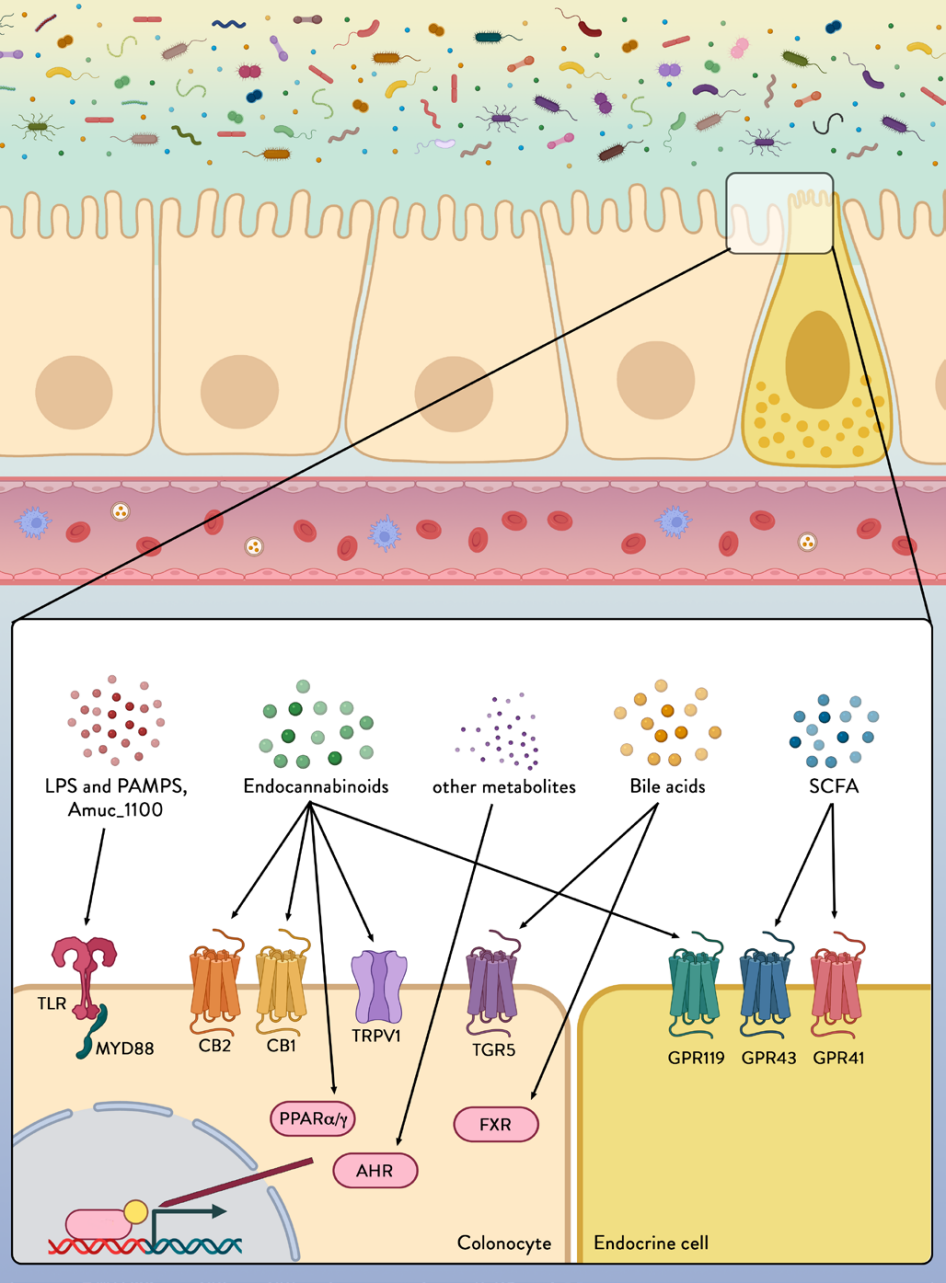
Colonocytes and endocrine cells express a variety of receptors able to sense and transmit signals from the microbial environment. Microbial/Pathogen-associated molecular patterns (PAMPs), lipopolyscaccharides (LPS) from the microbiota are detected by pattern recognition receptors, including toll-like receptors (TLRs). Amuc_1100 is a protein expressed on the outer membrane of *Akkermansia muciniphila* and which has been shown to signal through TLR2 to improve gut barrier function and reduce inflammation. Metabolites secreted by certain microbes (eg, endocannabinoids (eCBs)), generated by microbial digestion of dietary components (eg, short chain fatty acids (SCFAs)) or by transformation of host-derived factors (eg, eCBs and bile acids) can be sensed through various receptors and pathways to alter intestinal integrity and host health. CB1, CB2, cannabinoid receptor type 1 and type 2; TRPV1, transient receptor potential cation channel subfamily V member 1; FXR, farnesoid X receptor; AhR, aryl hydrocarbon receptor; GPR119, GPR43, GPR41, G-protein coupled receptor 119, 43 and 41; MYD88, myeloid differentiation primary response 88; PPARα/γ, peroxisome proliferator-activated receptors alpha and gamma; TGR5, Takeda G protein-coupled receptor 5.

SCFAs stimulate the secretion of gut peptides by acting on specific G-protein-coupled receptors expressed at the surface of the enteroendocrine L-cells that are specifically abundant in the terminal ileum and colon. These receptors, named G protein-coupled receptor (GPR)43 (or free fatty acid receptor 2 (FFAR2)) and GPR41 (or FFAR3) (figure 4),^73^ are also expressed in a wide variety of tissues and cell types (eg, adipocytes, immune cells).^74–76^ The key role played by the microbiome on the secretion of the gut peptides has been elucidated by using mouse models lacking either GPR43 or GPR41. Mice lacking these receptors exhibit reduced secretion of GLP-1 and PYY after exposure to SCFAs or specific prebiotics (figure 3).^77–79^

In addition to their well-known role and mechanisms of action, some SCFAs might also exert different functions from what has previously been thought. For example, butyrate has been described many times as an essential energy source for the colonic cells to proliferate and maintain the gut barrier. However, recent evidence shows that butyrate also strongly influences the microbial environment by communicating with colonic cells. Indeed, the abundance of oxygen in the luminal part of the gut and its gradually decreasing concentration towards the epithelium is a key requirement for anaerobic bacteria to remain in the close vicinity of the epithelium as opposed to facultative anaerobes such as Enterobacteriaceae (phylum Proteobacteria) that have been shown to increase the risk of intestinal inflammation (figure 3).^80 81^ Butyrate contributes to control of the anaerobic condition in the colon by activating the β-oxidation in the mitochondria. By activating the nuclear receptor peroxisome proliferator-activated receptor gamma (PPARγ) in the colonic cells, butyrate limits the diffusion of oxygen from the colonocytes to the luminal part, thereby maintaining the anaerobic conditions. The activation of PPARγ also represses the expression of the gene encoding inducible nitric oxide synthase, thereby reducing NO production and ultimately luminal nitrate levels that are specific energy sources used for the proliferation of putative pathogenic facultative anaerobes (Enterobacteriaceae) (figure 3).

Similar observations have been done in humans with severe intestinal inflammation, such as during IBD, cancer, obesity and diabetes,^52 82–84^ in which an increased abundance of Enterobacteriaceae has been detected.

Strikingly, numerous papers are pointing to the fact that all these diseases are also associated with a decreased abundance of bacteria that produce SCFAs, mostly propionate and butyrate.^85^ This is the case for instance of the bacteria *F. prausnitzii, A. muciniphila* and more recently *Dysosmobacter welbionis*.^86^ A specific case is made for *Anaerostipes* and *Anaerobutyricum* spp that generate butyrate from lactate in the presence of acetate using the acetyl-CoA pathway.^87^ These can form trophic chains in the small intestine as well as the colon where a variety of bacteria produce lactate. Accumulation of lactate, an undesired acid, in the gut environment results in GI disorders, potentially explaining some benefits of interventions with butyrogenic *A. soehngenii* in metabolic syndrome subjects.^32 88^

Besides propionate and butyrate, the impact of succinate is also being investigated. Succinate is best known as an intermediate of the Krebs cycle and considered as a substrate for mitochondrial oxidative phosphorylation, but is also a metabolic product from bacteria. In this context, succinate has been classically ignored because it was considered as being mainly a key intermediate in propionate synthesis. Currently, the role of succinate remains largely a matter of debate^89^ as there have been reports of both beneficial and opposite associations between succinate and insulin resistance, obesity and inflammation.^90–93^

### Lipopolyscaccharides/Pathogen-associated molecular patterns

The gut barrier is a complex and dynamic collection of physical and chemical structures that surveil the environment and protects the host from microbial invaders and harmful stimuli. Some of these hazardous components coming from the environment are the so-called pathogen-associated molecular patterns (PAMPs), of which bacterial LPS are the prototypical class.^94^

LPS, endotoxins found on the cell membranes of Gram-negative bacteria, are potent activators of the inflammatory response and release of even small amounts of LPS into the circulation are sufficient to elicit an inflammatory response.

LPS and other PAMPs exert their activity through activation of specific pattern recognition receptors (PRRs) that sense microorganisms and infectious agents and signal a defensive response. There are four major subfamilies of PRRs: the toll-like receptors (TLRs), the nucleotide-binding oligomerisation domain-leucine-rich repeats (LRR)-containing receptors, the retinoic acid-inducible gene 1 (RIG-1)-like receptors (aka RIG-1-like helicases) and the C-type lectin receptors.^95^ Among those the TLRs, a family of receptors comprising 10 members in human (13 in mice), are the best characterised. Each of the TLRs mediates responses to distinct microbial components derived from pathogens. Two typical examples are TLR2, which senses bacterial lipoproteins,^96^ and TLR4, which recognises bacterial LPS.^97^ Together the TLRs cover a wide range of both external stimuli (PAMPs)^98^ and internal signals derived from tissue damage (damage-associated molecular patterns (DAMPs)) (figures 3 and 4).^99^ These ligands come in many forms and sizes: from nucleic acids to lipids, from small compounds to macromolecules. TLRs are widely distributed in immune cells including macrophages, neutrophils, dendritic cells, natural killer cells, mast cells, basophils and eosinophils,^99^ but also in other body cells, such as intestinal epithelial cells. Their activation induces antigen-presenting cell activation, thereby bridging the innate and the adaptive immune responses, and stimulates signalling cascades as an attempt to fend off microbial invaders or repair the damaged tissue. Although this inflammatory response is required to eliminate the infection, excessive activation of TLRs can lead to disruption of immune homeostasis and the sustained pro-inflammatory cytokines and chemokine production can increase the risk of inflammatory diseases and autoimmune disorders. This is the case in metabolic endotoxaemia, in which high-fat diet and weight gain have been associated with a higher gut permeability and subsequent systemic (mild) elevation in circulating plasma LPS.^100^ This causes a state of low-grade inflammation, which is a pathological feature of a range of chronic conditions including T2D, NAFLD, chronic kidney disease and atherosclerosis.^38 39 101 102^ Interestingly, LPS from different types of bacteria have distinct effects on gut-barrier function, adipose inflammation, intestinal glucose absorption, blood glucose, insulin and incretins, indicating that the net effect of metabolic endotoxaemia levels on host metabolism can vary in function of gut microbiota composition.^103^

Disruption of PRRs expression has been associated with alterations in the microbiota composition that favour inflammation. For example, mice deficient in TLR5, which is activated by bacterial flagellin, develop colitis or metabolic syndrome, associated with an altered microbiota.^104 105^

The TLR activation of downstream signalling pathways has been shown to be dependent on myeloid differentiation factor 88 protein (MyD88) (figure 4). MyD88 is an essential adaptor protein for all TLRs, except TLR3^106^ and deletion of MyD88 in the intestines partially protects against diet-induced obesity, diabetes and inflammation and increases anti-inflammatory endocannabinoids (eCBs), restores antimicrobial peptides production and increases intestinal regulatory T cells during diet-induced obesity.^107^

Although many questions remain to be answered before we have a full understanding of how PAMS/DAMPS, PRRs, the microbiome and disease state interact, our growing understanding of this complex interplay is opening new therapeutic possibilities for inflammation-dependent disorders.

### Bioactive lipids

#### Endocannabinoid system

Over the last two decades, the eCB system has been widely explored because of its extensive range of physiological effects. Among its pleiotropic effects, the eCB signalling system appears to play a key role in regulating energy, glucose and lipid metabolism but also in immunity, inflammation and more recently in microbiota-host interactions.^108^ ^109^

Historically, it was in 1988 that the first endogenous cannabinoid receptor type 1 (CB_1_) was identified as being activated by the psychoactive compound of *Cannabis sativa*, Δ9-tetrahydrocannabinol,^110^ followed by the discovery of a second receptor in 1993, the cannabinoid receptor type 2 (CB_2_).^111^ Both receptors are GPRs and share common signalling mechanisms.^112^ The first endogenous agonist identified was anandamide (*N*-arachidonoylethanolamide (AEA)). AEA is one of the key members of a large group of bioactive lipids that belong to the *N*-acylethanolamine (NAE) family.^113^ The second key ligand identified was 2-arachidonoylglycerol (2-AG).^114^ Since the discovery of these two major compounds, the eCB family has been expanded and is no longer restricted to only eCBs with specific activity on CB_1_ and CB_2_ receptors. For example, some eCBs also interact with PPARα and PPARγ, as well as with other membrane receptors such as GPR55, or transient receptor potential vanilloid type-1 (TRPV1) (figure 4). Besides the so-called ‘true’ eCBs, that is, eCBs able to bind CB_1_ and CB_2_, numerous other compounds with structural resemblance to the prototypical eCBs have been shown to interfere with the eCB response without directly activating CB_1_ or CB_2_ eCB receptors. All these molecules are referred to as eCB-like compounds or congeners and are bioactive lipids including other NAEs or members of the acylglycerol family.^115 116^ However, eCB-like compounds can also exert pharmacological activity of their own. For example, *N*-oleoylethanolamine (OEA) or *N*-palmitoylethanolamine (PEA) can activate PPARα and TRPV1, and OEA, *N*-linoleylethanolamine (LEA) and 2-oleoylglycerol (2-OG) are able to activate GPR119.^117^ More recently, it was shown that both 1-palmitoylglycerol (1-PG) and 2-palmitoylglycerol (2-PG) are PPARα agonists (figure 4).^118^

In 2010, it was discovered that among the metabolic systems involved in the regulation of the gut barrier function the eCB system was playing a major role.^108^ It began with the finding that the intestinal eCB system is altered during obesity and diabetes, with an increased abundance of AEA that triggers gut permeability via CB_1_-dependent mechanisms.^108^ Interestingly, this modification of the eCB system tone was associated with changes in the gut microbiota. Moreover, pharmacological activation of the eCB system with a potent eCB agonist increased adipogenesis and disrupted the gut barrier.^119^ In a series of independent studies, the link between gut microbiota, adipose tissue metabolism and the eCB system has been confirmed since both genetically obese and diabetic mice (*ob/ob* and *db/db*) present a profound shift in their gut microbiota composition, which is associated with altered whole body tissue metabolism and eCB system tone.^120 121^ Taken together, these data strongly support a link between specific bioactive lipids, belonging to the eCB system, and the gut microbiota, the development of the adipose tissue and intestinal function.

To further explore the underlying mechanisms and to demonstrate whether the synthesis of these NAEs could be involved in the onset of metabolic disorders and changes in the gut microbiota, several mouse models have been generated in which *N*-acylphosphatidylethanolamine-hydrolysing-specific phospholipase D (NAPE-PLD), a key synthesis enzyme, has been inactivated in either adipocytes, intestinal epithelial cells or hepatocytes.^122–125^

Mice lacking NAPE-PLD in adipocytes spontaneously developed obesity, insulin resistance and inflammation on a normal caloric diet and were more sensitive to high-fat diet-induced metabolic disorders.^122^ The adipocyte-specific deletion of NAPE-PLD decreased the thermogenic programme (ie, browning/beiging) in adipose tissue and resulted in a profound shift in the gut microbiota composition. Moreover, transferring the microbiota from adipose tissue NAPE-PLD deleted mice to germ-free recipient mice replicated the overall phenotype,^122^ suggesting a causal role of the gut microbiota. When deleting NAPE-PLD in intestinal epithelial cells, a different phenotype occurred. Mice became hyperphagic on first exposure to a high-fat diet and then developed exacerbated diet-induced obesity and hepatic steatosis. Mechanistically, this was attributed to a defect in the gut-to-brain axis, as hypothalamic Pomc neurons alterations were found, likely explained by changes in both intestinal and plasma eCBs. Strikingly, the gut microbiota was also affected in this model and modulating the microbiota could partially revert the phenotype.^123^ In the last model, mice deleted for the NAPE-PLD in the hepatocyte developed a high-fat diet-like phenotype under normal diet (ie, increased fat mass gain, hepatic steatosis, liver inflammation). These effects were related to changes in other key bioactive lipids known to be influenced by the gut microbiota such as BAs.^124^ Collectively, all these data and animal models suggest that the eCB system, through the NAPE-PLD, is dialoguing with the gut microbiota via the production of bioactive lipids, and it turn any dysregulation of this enzyme can lead to metabolic complications.

To further explore the potential links between the gut microbiota and the regulation of the eCB system, the endocannabinoidome (eCBome) of germ-free mice was compared with that of conventionalised mice at different time-points. The eCBome is an extension of the eCB system that comprises over 50 receptors and metabolic enzymes, and >20 lipid mediators with important functions.^126^ An age-dependent modification in intestinal eCBome gene expression and lipid mediator levels was found. Strikingly, faecal material transplantation from control mice donors to age-matched germ-free mice reversed several of these alterations, already after only 1 week.^126^ Altogether, this set of studies demonstrate that the gut microbiota is directly impacting the host eCBome.

In conclusion, all evidence points towards a bidirectional cross-talk between the host’s eCB system and the gut microbiota. However, further investigations are warranted to untangle the many remaining mysteries of this relationship. Adding to the complexity, it has recently been shown that the gut microbiota itself is able to produce specific eCBs.^127^ This opens new exiting opportunities of exploring the microbiota to host interaction and offers several novel putative targets for therapy.

#### Bile acids

Primary BAs, such as cholic acid (CA) and chenodeoxycholic acid (CDCA) in humans (and muricholic acid (MCA) in rodents), are amphipathic molecules synthesised in the liver from cholesterol.^128^ They can be conjugated to glycine or taurine prior to their secretion into bile and storage in the gallbladder. When food is ingested, BAs are released into the small intestine where they assist in the digestion and absorption of dietary fat. Around 95% of intestinal BAs are reabsorbed in the ileum to return to the liver for re-secretion. This enterohepatic circulation of BAs occurs several times a day and is an important physiological mechanism for maintaining whole body glucose, lipid and energy homeostasis to prevent hyperglycaemia, dyslipidaemia and obesity, and it protects against inflammatory metabolic diseases of the digestive and cardiovascular systems.^129^ Only a small fraction of the BAs will escape this highly efficient loop and reach the colon. These BAs are then either reabsorbed passively into the circulation or excreted via the faeces. The losses in BAs are compensated by de novo hepatic synthesis, which is regulated by fibroblast growth factor-19 (FGF19) in humans signalling in the small intestine (FGF15 in rodents).

Although the primary function of BAs is to regulate the digestion and absorption of cholesterol, triglycerides and fat-soluble vitamins, it has been recently recognised that BAs also serve an endocrine function as they act as signalling molecules. Moreover, BAs have been shown to modulate epithelial cell proliferation, gene expression, lipid, glucose and energy metabolism by activating several receptors. These receptors are the vitamin D receptor,^130^ pregnane X receptor,^131^ constitutive androstane receptor,^132^ farnesoid X receptor and G-protein-coupled bile acid receptor-1 (also known as Takeda G protein-coupled receptor 5 (TGR5)) (figure 4). These receptors are present in numerous tissues including the liver, intestine, muscle, brown adipose tissue and central and peripheral nervous systems and mediate the signalling cascade and activate expression of genes involved in the metabolism of BA, lipids and carbohydrates and in energy expenditure and inflammation. Signalling through FXR and TGR5 receptors has also been linked to the secretion of GI hormones such as PYY and GLP-1 (figures 3 and 4), known to be integral to the maintenance of energy and metabolic homeostasis. The role of BAs in the control of glucose, lipid and energy metabolism has been reviewed previously^128 133 134^ and will therefore not be reviewed in detail here.

Primary BAs are susceptible to be modified by gut microbes all along the intestinal tract. These modifications include deconjugation (the removal of amino acid residues) via bile salt hydrolase (BSH) activity and further metabolisation via removal of hydroxyl groups (dehydroxylation), oxidation (dehydrogenation) or epimerisation.^135 136^ This results in the formation of secondary BAs such as deoxycholic acid, lithocholic acid and ursodeoxycholic acid (a secondary BA in humans, although a primary BA in rodents). This bacterial metabolism changes the bioavailability and bioactivities of BAs, and consequently their impact on the metabolic responses they are involved in.^137^ Because of their signalling capacities and the fact that BAs are chemically transformed by the gut microbiota, BAs can therefore be considered as microbiota-derived signalling metabolites. Interestingly, there is a spatio-temporal pattern to be recognised as BAs are released after food intake and then encounter different microbial communities along the intestinal tract. While BSH activity can be carried out by a wide variety of bacteria distributed among many phylogenetically different bacterial divisions, including species able to colonise the small intestine,^138^ the other reactions are thought to be more restricted to more specialised bacterial species that reside in the distal part of the gut. Therefore, to fully understand the role of the gut microbiota on host metabolism, it is essential to study the involvement of the different bacteria capable of converting BAs. A recent study in centenarians suggested that their specific gut microbiota signatures may partially account for their decreased susceptibility to ageing-associated illnesses, chronic inflammation and infectious diseases by generating unique secondary BAs. This implies that manipulating the BA pool via modulation of the gut microbiota composition could represent a feasible way to combat diseases.^139^

### Aryl hydrocarbon receptor: a link to energy metabolism, inflammation and gut microbiome

The aryl hydrocarbon receptor (AhR) is expressed ubiquitously in vertebrate cells and this transcription factor is activated after ligand binding. Numerous AhR ligands exist including environmental triggers, nutrition-derived signals, various phytochemicals and bacterial metabolites such as tryptophan (figure 2). AhR ligand binding results in translocation of the AhR into the nucleus where it is bound to its dimerisation molecule AhR nuclear translocator resulting in the transcription of numerous genes involved in immunity and inflammatory processes (figure 3). Bacterial products and metabolites play a key role as activators and therefore several reports from the past years have tried to figure out the interplay of AhR with the gut microbiota (figures 3 and 4).^48 140^ Importantly, this AhR pathway has also been linked to energy metabolism and metabolic syndrome as there exists a reduced capacity both in preclinical and clinical settings of metabolising tryptophan into AhR binding derivates in metabolic syndrome.^141^ These authors showed that an increase of AhR ligands achieved by the administration of a *Lactobacillus* strain improved metabolic functions paralleled by ameliorated intestinal barrier and reduced hepatic steatosis. Indigo, a naturally occurring AhR ligand with potent anti-inflammatory activities, protects against high-fat diet-induced obesity and metabolic disturbances by upregulation of *Lactobacillus* spp and the key barrier cytokines interleukin (IL)-10 and IL-22.^142^ Microbial tryptophan metabolites such as indole-3-ethanol, indole-3-pyruvate and indole-3-aldehyde protect the gut epithelial barrier by affecting the integrity of the apical junctional complex including myosin IIA and ezrin.^143^ In experimental alcoholic liver disease, a disease where the gut microbiome is substantially impaired,^144^ induction of AhR ligands and administration of 6-formylindolo (3,2-b) carbazole (Ficz) improved alcoholic liver disease.^145^ Caspase recruitment domain family member 9^−/−^ mice are more susceptible to colitis and their microbiota fail to metabolise tryptophan to its respective metabolites.^146^ Transfer of this microbiota into wild-type mice increases colitis and can be improved by treatment with *Lactobacillus* strains delivering high amounts of AhR ligands. The AhR pathway might have also major implications for other inflammatory GI disorders such as coeliac disease.^147^ Patients with active coeliac disease show reduced AhR ligand production in their gut compared with non-coeliac control subjects and furthermore in non-obese diabetic mice expressing DQ8 (a transgenic mice that carry only human MHC class II DQ8), a high-tryptophan diet, treatment with *Limosilactobacillus reuteri*, a bacterial strain producing large amounts of AhR ligands or treatment with the AhR ligand Ficz decreased intestinal pathologies after gluten exposure.^147^ Germ-free mice show an impaired differentiation and repair of the epidermal barrier and mice lacking AhR in keratinocytes specifically are highly susceptible to cutaneous infections and barrier damage and colonisation with a defined group of bacteria restored the barrier. It remains to be established in this model which role intestinal bacteria might play in a putative gut-skin axis.^148^ The AhR pathway therefore reflects a prototypic pathway at the interface microbiota-epithelial barrier-metabolism and immune functions.

### Key bacteria and their specific molecules

Most signalling metabolites can be produced by large numbers of different gut bacteria, and hence have limited specificity. However, various bacteria can make specific molecules that have unique interactions with the host (figures 2–4). For obvious reasons, these have been very well characterised in pathogens that can make specific toxins, synthesise polysaccharides to evade the immune system or induce the host to synthesise receptors, allowing them to invade. However, recent research has identified new and unique host signalling molecules that are found in potentially symbiotic gut bacteria. These include immunomodulatory polysaccharides and sphingolipids produced by *Bacteroides* spp^149 150^ and muropeptides formed by *Enterococcus* spp.^151^

A special class of unique molecules formed are proteins that are genetically encoded by one or a few strains of the same species. Some have been studied in detail and often involve stable or post-translationally modified proteins that have the potential to interact with host receptors as they are secreted, or cell envelope located. Some of these derived from bacteria that are already widely marketed as probiotics, including *L. acidophilus* NCFM that produces a large, likely glycosylated surface layer protein signalling to the DC-SIGN receptor,^152^ the 90 kDa pilus protein SpaC of *L. rhamnosus* GG that is a partly glycosylated mucus-binding protein with unusual signalling capacity to DC-SIGN receptor on dendritic cells^152 153^ and the pilus-located Tad protein found in some *Bifidobacterium* spp that promotes colonic persistence and epithelial proliferation.^154 155^ A recently studied protein is the caseinolytic protease B (ClpB) of *E. coli* that is an antigen-mimetic of alpha-melanocyte-stimulating hormone and increases satiety via increased plasma GLP-1 and PYY production.^156^ ClpB proteins are well-known moonlighting proteins found to be partially secreted by a variety of bacteria, including Lactobacilli and Bifidobacteria. The specificity of ClpB, however, may be not so high as a ClpB-producing *Hafnia alvei* was also found to suppress satiety to some extent in a human trial.^157^

Considering the effectiveness of live and pasteurised *A. muciniphila* administration in a proof-of-principle human trial,^43 118 158^ it is not surprising that several proteins from *A. muciniphila* have recently been identified with potential signalling capacity. A recent one is an 84 kDa protein (encoded by the Amuc_1831 gene) termed P9, which after oral administration was found to induce serum GLP-1 in mice. In vitro studies indicated that P9 interacts with the intercellular adhesion molecule 2 receptor.^159 160^ Another recently discovered protein identified is the *A. muciniphila* 50 kDa Amuc_1434* protein that was found to suppress LS174T cell viability via tumour-necrosis-factor-related apoptosis-inducing ligand (TRAIL)-mediated apoptosis pathway.^161^ However, both of these proteins are found in many bacteria other than *A. muciniphila*, both are annotated as proteases suggesting enzymatic activity, and both have not been localised outside the cells, not excluding the possibility that cell lysis is needed for their activity. Even more importantly, their stability has not been addressed, which is of interest as pasteurised *A. muciniphila* cells were as effective or even more than live cells both in human and mice models.^43 162 163^ All these arguments do not apply to the other *A. muciniphila* protein that was discovered to be signalling to TLR2 (figure 4).^163^ This is the 30 kDa Amuc_1100 protein that has been defined as an outer membrane protein with virtually no homology to other bacteria outside the Verrucomicrobia and in fact is suggested to be a pilus-associated protein.^164^ Further studies showed Amuc_1100 to be thermostable and preventing diet-induced obesity in a mouse model.^163^ When comparing all three proteins for their absolute abundance, it is evident that Amuc_1100 is much more abundant than the other two signalling candidates in proteomes of *A. muciniphila* grown on mucin.^165^ Hence, future comparative studies should determine which proteins or combinations thereof can explain the observed activity of *A. muciniphila* in humans.

### Newly identified molecules, impact on health and their targets

Besides the classical molecules such as SCFAs, BAs or PAMPs and gut peptides (ie, GLP-1, PYY), all described as regulators of the host metabolism, gut barrier and inflammation, the role of a novel class of molecules called ‘enterosynes’ is emerging. The concept of enterosynes has been recently introduced and defined as ‘*molecules originating from the gut which have the capacity to modulate duodenal contraction by targeting the enteric nervous system (ENS). Enterosynes can be chemically diverse and related to hormones, bioactive peptides/lipids, nutrients, microbiota and immune factors’*.^166^

The origin of this concept is based on the observation that subjects with T2D are characterised by a duodenal hypermotility which favours glucose absorption and contributes to hyperglycaemia.^166 167^ It has been demonstrated that duodenal contractions are sensed by the hypothalamus^167–170^ and that during diabetes the duodenal hypermotility creates aberrant afferent nervous messages to the brain.^169 171^ Conversely, restoring natural duodenal contraction by acting on ENS neurons restores the gut-brain axis and improves insulin sensitivity.^166^

The connections between gut microbiome, brain function and glucose metabolism are becoming a hot topic in this area of research and the role of the ENS emerged as a new target to tackle diseases such as diabetes. Although various papers are discussing strategies to modulate the gut microbiome, such as probiotics, prebiotics and faecal transplants, in view of alleviating features of metabolic syndrome, few, if not none, of them are characterising intestinal actors such as enterosynes.

In search of novel gut molecules and receptors involved in glucose metabolism, the action of specific fibres known to change the gut microbiota and improve diabetes was explored. The administration of oligofructose decreased duodenal contraction frequency by controlling enteric neurons activity. This led to reduced hyperglycaemia and decreased inflammatory markers in the adipose tissue of the diabetic mice.^170^ By using lipidomic analysis, it was discovered that this oligofructose feeding selectively increased the abundance of an intestinal bioactive lipid (12-hydroxyeicosatetraenoic acid (12-HETE)) in the colonic cells. Strikingly, the administration of 12-HETE to diabetic mice improved glucose metabolism. The effect of 12-HETE was also confirmed ex vivo. Furthermore, they discovered that the molecular mechanism by which this bioactive lipid acts on duodenal contractility is dependent on the presence of the mu-opioid receptors (MOR) (activated by enkephalin) and PPARγ. The preclinical findings were supported by human data showing a reduction in the levels of 12-HETE and a decreased expression of the proenkephalin and MOR in the duodenum of patients with diabetes as compared with healthy subjects.^170^

Using various approaches with dietary supplements to tackle IBDs, new bioactive lipids with anti-inflammatory properties were identified.^172^ Exploiting mass spectrometry of the *E. coli* Nissle 1917 (EcN), a well-studied strain marketed as a probiotic for the treatment of colitis, led to the discovery that the concentration of 3-hydroxyoctadecaenoic acid (C18-3OH) was increased. They found that oral administration of C18-3OH decreased colitis. To determine whether other bacteria present in the gut microbiota produce C18-3OH, the gut microbiota was modulated by using oligofructose. The authors found that the anti-inflammatory properties of oligofructose were associated with an increase in colonic C18-3OH concentration. Finally, they identified specific bacteria producing this bioactive lipid and discovered that C18-3OH acts by activating PPARγ.^172^

Altogether, these two examples show that the gut microbiota is the source of putative numerous bioactive compounds (figure 2) acting on host receptors involved in the regulation of metabolism and inflammation (figure 3).

Whereas some metabolites are desired for health, others may be harmful, but evidence only derives from association studies or animal testing. Three metabolites with negative impact have been subject to recent studies, including fructoselysine, an advanced glycation end product (AGE), trimethylamine N-oxide (TMAO) and imidazole propionate (IMP).

AGEs are Maillard reaction products formed in our foods by thermal processing when free amino groups of proteins and amino acids react with reducing carbohydrates, forming compounds that are poorly bioavailable. A body of mechanistic evidence has linked AGEs to T2D and CRC through stimulation of the pro-inflammatory response via the activation of the receptor of AGEs,^173 174^ an increase in gut permeability—allowing closer interaction of AGEs with colonic epithelium—and consequential leakage of bacterial toxins into the systemic circulation.^175^ Fructoselysine is an Amadori product formed from lysine and glucose that is one of the primary dietary AGEs. Earlier studies showed that *E. coli* has the capacity to respire fructoselysine.^176^ However, recent analysis showed that it can be converted into butyrate by members of the genus *Intestinimonas* spp via a novel pathway.^70^ Of note, the capability to degrade fructoselysine was experimentally and computationally only observed in formula-fed but not in breast-fed infants, which may relate to high contents of this compound in formulas after thermal treatment.^177^ Further studies should address the causality of fructoselysine and other AGEs in T2D and other diseases and the involvement of intestinal bacteria in their conversion. A recent study also described the complete utilisation of N-ε-carboxymethyllysine, another major AGE, by *Cloacibacillus* and potentially *Oscillibacter* spp.^178^

TMAO is related to the intake of quaternary amines, such as betaine, choline and L-carnitine, which are commonly found in vegetables, fruits, meat and seafood and are well-known bacterial osmoprotectants.^179 180^ Several gut bacteria including several Proteobacteria can convert these quaternary ammonium ions via TMA lyase and its activating enzyme (CutCD) into acetaldehyde and trimethylamine (TMA). TMA then can enter the bloodstream and is converted by flavin monooxygenase in the liver to TMAO. Recent studies have shown that TMAO in serum is strongly associated with atherosclerosis and cardiovascular risks.^181^ In addition, TMAO has been shown to promote formation of atherosclerotic plaques in a mouse model.^182^ Moreover, TMAO is a common uraemic toxin.^183^ Hence, there is considerable interest in understanding the metabolism of these quaternary ammonium ions into compounds that do not lead to TMA or other TMAO precursors. New insight has come from the biochemical and pathway analysis of *Eubacterium limosum* and its related gut isolate *E. maltosivorans* that were found to deaminate betaine and other quaternary amines in a novel process involving bacterial cell compartments and leading to the production of acetate and butyrate.^184–186^ While the latter bacteria are highly related and appear to have a unique vitamin B_12_-dependent metabolic pathway, this does not hold for the metabolism of histidine that may lead to the production of IMP. It has been shown that concentration of IMP was increased in the serum of patients with T2D.^187^ It was recently found that IMP is produced from histidine by intestinal bacteria that impaired insulin signalling and glucose tolerance through the mammalian target of rapamycin complex 1-dependent pathway.^188^ Two unrelated bacteria, *Streptococcus mutans* and *Eggerthella lenta* have been identified as IMP producers, confirming the fact that many metabolites are produced by several groups of intestinal bacteria that may not share any phylogenetic relations. Altogether, these examples indicate the involvement of intestinal bacteria in generating undesired compounds but identified also new anaerobes that may detoxify these and even convert these in products such as butyrate that have beneficial signalling potential (figures 2 and 3).

## General conclusion and perspectives

Over the last two decades, considerable progress has been achieved. From initial clinical observations to more mechanistic approaches, the field of gut microbiota and health is evolving to irrefutable causal links. However, there are still numerous studies that claim causality when in fact only correlations are being demonstrated. Moving from correlation to causality remains an important and required step to better design putative interventions based on the modulation of the gut microbiota or by using specific active compounds.^50 189^ Thanks to the numerous efforts and the advance in omics analysis, the scientific community is gradually moving towards personalised medicine and the microbiome era is clearly an important part of the paradigm shift in the future of medicine and nutritional approaches.
